# A review on phytochemical and pharmacological facets of tropical ethnomedicinal plants as reformed DPP-IV inhibitors to regulate incretin activity

**DOI:** 10.3389/fendo.2022.1027237

**Published:** 2022-11-11

**Authors:** Srishti Chhabria, Shivangi Mathur, Sebastian Vadakan, Dipak Kumar Sahoo, Pragnyashree Mishra, Biswaranjan Paital

**Affiliations:** ^1^ Department of Biochemistry and Biotechnology, St Xavier’s College, Ahmedabad, India; ^2^ Department of Biotechnology, Gujarat University, Ahmedabad, India; ^3^ Department of Biotechnology, President Science College, Ahmedabad, India; ^4^ Department of Veterinary Clinical Sciences, College of Veterinary Medicine, Iowa State University, Ames, IA, United States; ^5^ Department of Horticulture, College of Agriculture, Odisha University of Agriculture and Technology, Chipilima, Sambalpur, India; ^6^ Redox Regulation Laboratory, Department of Zoology, College of Basic Science and Humanities, Odisha University of Agriculture and Technology, Bhubaneswar, India

**Keywords:** dipeptidyl peptidase-IV, insulin resistance, incretin, polyherbal formulations, antioxidants, hormonal disorder

## Abstract

Type 2 diabetes mellitus is a metabolic disorder resulting from impaired insulin secretion and resistance. Dipeptidyl peptidase (DPP)-IV is an enzyme known to trigger the catalysis of insulinotropic hormones, further abating the endogenous insulin levels and elevating the glucose levels in blood plasma. In the field of drug development, DPP-IV inhibitors have opened up numerous opportunities for leveraging this target to generate compounds as hypoglycemic agents by regulating incretin activity and subsequently decreasing blood glucose levels. However, the practice of synthetic drugs is an apparent choice but poses a great pharmacovigilance issue due to their incessant undesirable effects. The ideology was set to inventively look upon different ethnomedicinal plants for their anti-diabetic properties to address these issues. To date, myriads of phytochemicals are characterized, eliciting an anti-diabetic response by targeting various enzymes and augmenting glucose homeostasis. Antioxidants have played a crucial role in alleviating the symptoms of diabetes by scavenging free radicals or treating the underlying causes of metabolic disorders and reducing free radical formation. Plant-based DPP-IV inhibitors, including alkaloids, phenolic acid, flavonoids, quercetin, and coumarin, also possess antioxidant capabilities, providing anti-diabetic and antioxidative protection. This review article provides a new gateway for exploring the ability of plant-based DPP-IV inhibitors to withstand oxidative stress under pathological conditions related to diabetes and for reforming the strategic role of ethnomedicinal plants as potent DPP-IV inhibitors through the development of polyherbal formulations and nanophytomedicines to regulate incretin activity.

## Introduction

**Figure 1:**
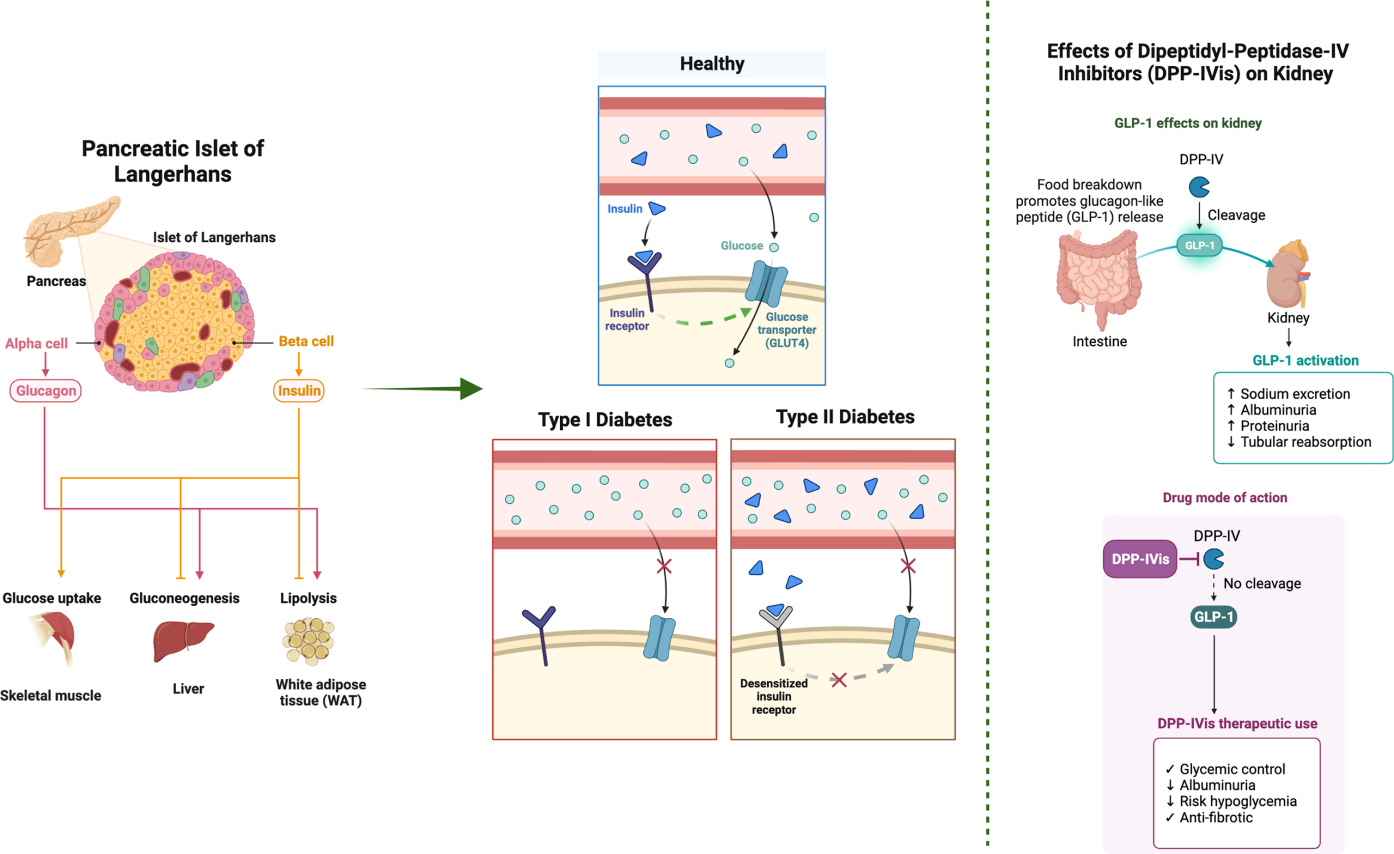
An overview of the impact of dipeptidyl peptidase (DPP)-IV inhibitors on the kidney and the insulin signaling pathways. The key aspects of the insulin signaling pathway, such as insulin binding, signal cascade, exocytosis, and glucose entry, are presented. DPP-IV inhibitors (DPP-IVis) are non-insulin glucose-lowering agents that are members of the incretin family. They are able to improve glycemic control while maintaining an acceptable level of safety. In individuals with chronic kidney disease, these medications are associated with a minimal risk of hypoglycemia, no weight gain, and good overall tolerability. The results of clinical trials are still up for debate, even though several experimental and clinical research point to the possibility that DPP-IVis may exert considerable pleiotropic effects, particularly on the kidneys. The figure was created with BioRender.com.

Diabetes mellitus (DM) is a chronic hyperglycemic metabolic condition caused by decreased insulin production, peripheral insulin resistance, or both. According to a WHO study, diabetes was the ninth biggest cause of death in 2019, directly responsible for almost 1.5 million fatalities. Type 2 diabetes mellitus (T2DM) affects the majority of people with diabetes, accounting for more than 90% of those with diabetes, and is characterized by insulin secretion defects in pancreatic β-cells and insulin resistance, whereas type 1 DM is caused by autoreactive T cell-mediated destruction of β-cells (**Figure 1**) (1, 2). In reaction to food consumption, pancreatic β-cells secrete the hormone insulin. The primary function of insulin is to regulate blood glucose levels by inducing muscles, liver, and fat cells to absorb accumulated glucose from the bloodstream and store it as an energy source. T2DM, also called non-insulin DM, plays a cardinal role in the humongous populace and is triggered by an interplay of environmental and genetic factors (3). It is hallmarked by hyperglycemia and characterized by the impairment of the inositol triphosphate kinase (PI3K-Akt) pathway (metabolic arm) of insulin signaling, failing to transport glucose and synthesize glycogen and thus further leading to compensatory hyperinsulinemia to maintain euglycemia which ultimately causes insulin resistance (4, 5).

Consequently, this results in the redundant stimulation of the unaffected RAS-MAPK pathway (mitogenic arm) of insulin signaling, contributing to cardiovascular dysfunction and endothelial injury and advancing myriads of chronic diabetic complications by significantly compromising the quality of life (5). The previous studies show a growing prevalence of DM in India, triggering a shift in the onset age of DM from adult to adolescence (6). This invokes a constant need for a promising treatment to mitigate DM and the progression of chronic symptoms.

## Role of incretin hormones

The insulinotropic effects of incretin hormones are the reason why the body produces more insulin in response to meals when glucose is ingested orally rather than when it is administered intravenously, even when the plasma glucose levels are the same. This phenomenon is referred to as the incretin effect (7). The previous studies demonstrated that gut extracts possess a hormone that tightly regulates the secretion of pancreas and was named as glucose-lowering element or incretin (INtestine seCRETtion Insulin). It was verified that diabetic patients unveil a total loss of the incretin effect. Henceforth, a hypothesis suggesting that an impaired incretin function contributes to the pathogenesis of T2DM was formulated. The two most potent incretin hormones released in response to oral glucose are glucagon-like peptide-1 (GLP-1) (**Figure 1**) and gastric inhibitory peptide (GIP) (7–9).

GIP is a peptide of 42 amino acids belonging to the glucagon secretin family of peptides secreted from the K-cells of the upper intestine was earlier known to inhibit gastric acid secretion, so it was called gastric inhibitory peptide, but then later, it also showcased its efficacy on the pancreas by stimulating insulin secretion glucose dependently and was renamed as glucose-dependent insulinotropic peptide (7–9). GLP1 is the second most potent incretin hormone, a peptide of 31 amino acids known to be one of the enteroglucagons synthesized from proglucagon genes while secreted from both pancreatic alpha cells as well as L cells of the lower intestine and colon, which are known to stimulate the islets of the pancreas and secrete insulin (8).

Both GLP-1 and GIP validate their functional role by binding to their specific receptors GLP-1R and GIPR, which belong to the G protein-coupled receptor family triggering adenylate cyclase activity and elevating the levels of intracellular cyclic adenosine monophosphate (C-AMP) in pancreatic β-cells with the activation of protein kinase A (PKA) and exchange protein activated by C-AMP2 (EPAC2) involved in a broad range of intracellular actions such as altered ion channel activity, elevated cytosolic calcium levels which facilitate the fusion of insulin granules to the plasma membrane, and enhanced exocytosis of insulin-containing granules, contributing to the enhancement of insulin secretion in a glucose-dependent manner (8–10). Both GLP-1 and GIP induce insulin secretion in response to oral glucose consistently with their functional role as incretins. Based on data from several studies previously, both incretins share a few common insulinotropic actions, like increasing insulin secretion, enhancing resistance to apoptosis, and increased β-cell proliferation, but also differ in a few biological attributes, like GIP stimulates the pancreas by increasing glucagon secretion and acts on the gastric tract by inhibiting gastric acid secretion, whereas GLP 1 decreases glucagon secretion and acts on the gastrointestinal (GI) tract by decreasing gastric emptying and postprandial blood glucose (8, 10).

## Crux of the matter: Dipeptidyl peptidase-IV

**Figure 2:**
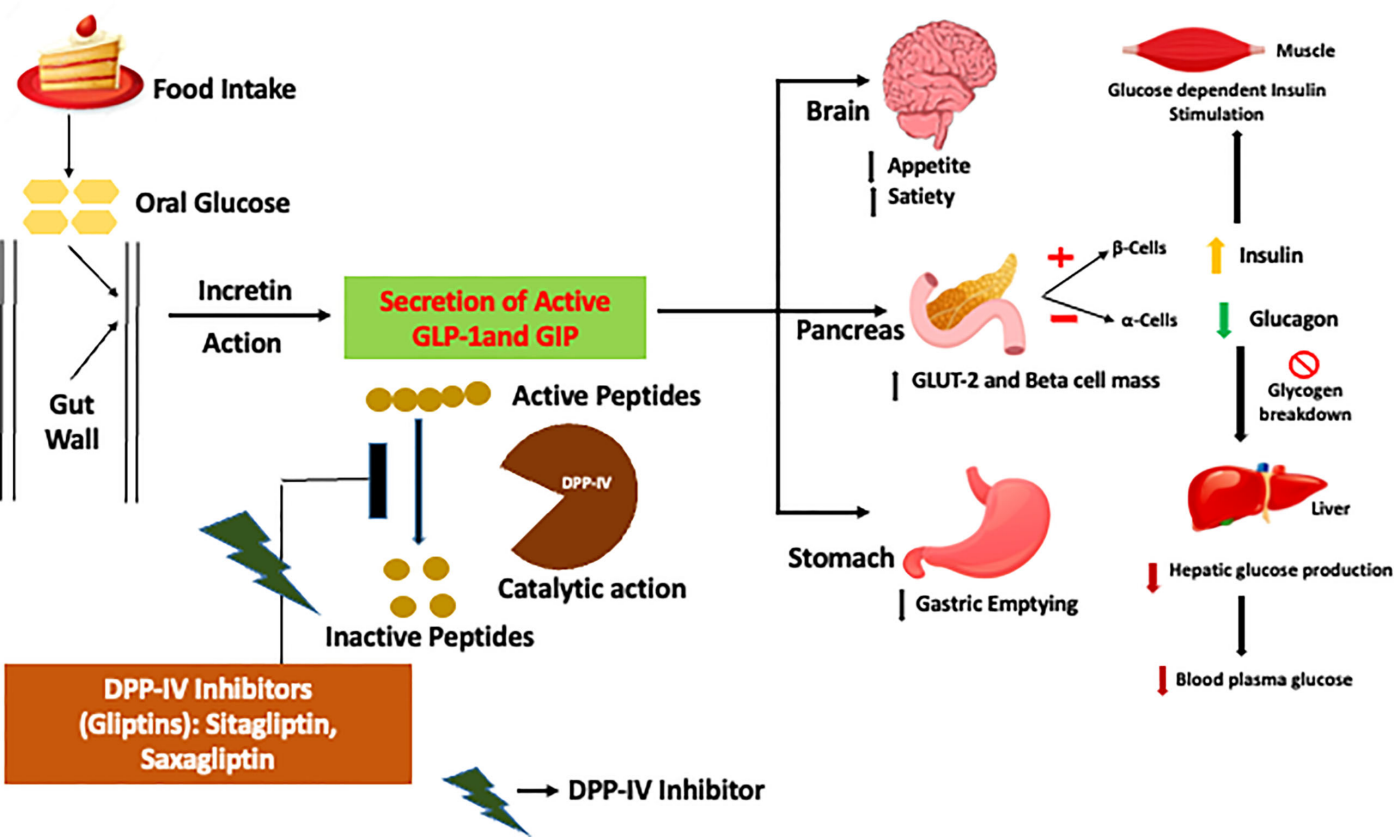
Model of the mode of action of dipeptidyl peptidase (DPP)-IV inhibitors for augmenting incretin activity. In healthy individuals, food intake aids the secretion of incretin hormones from the alimentary canal, which further favors the synthesis and release of insulin and additionally arrests the synthesis and release of glucagon, subsequently maintaining the glucose levels in blood plasma. However, type 2 diabetes mellitus patients incur a loss of incretin activity by the catalytic action of the DPP-IV enzyme, which is abated by DPP-IV inhibitors in order to escalate the incretin levels for regulating glucose homeostasis. .

The human gene dipeptidyl peptidase (DPP)-IV is located on chromosome 2 and encodes dipeptidyl peptidase IV, a serine protease, an enzyme located on epithelial as well as endothelial cells found to be expressed in varied tissues including the liver, gut, placenta, and kidney, causing the catalytic degradation and reduction in the half-lives of GLP-1 and GIP levels and further ensuing the perturbation of glucose homeostasis (**Figure 2**). From the recent investigations done, it was reported that 75% of GLP-1 metabolism by DPP-IV and elimination occurs in the gut and liver, respectively, permitting only 10–15% of GLP-1 circulation in the blood (8, 10). In order to augment the insulinotropic activity in blood plasma, various synthetic and herbal DPP-IV inhibitors have gained much focus as oral hypoglycemic agents with minimal side effects (7, 11, 12).

## Status of the naturally available DPP-IV inhibitors

**Figure 3:**
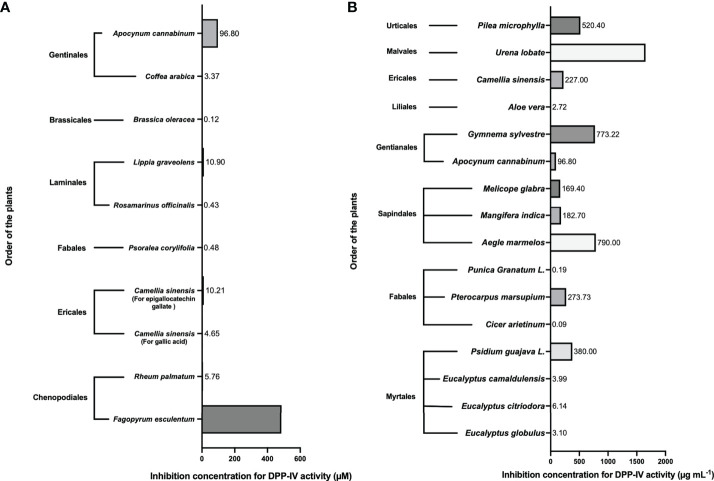
Levels of dipeptidyl peptidase (DPP)-IV activity inhibition concentration of bioactive compounds in certain plants studied. Different inhibitory concentrations of the plant products are found for the inhibition of DPP-IV (13–28). In **(A)**, the inhibitory concentration is evaluated in μM, while in **(B)** the inhibition concentrations are emulated in μg/ml.

Various naturally available plant-based compounds are investigated as DPP-IV inhibitors. Different plant parts such as leaves, seeds, flowers, buds, and bulbs are analyzed to identify their inhibitory capacity against DPP-IV activity. Parts of the plants were found to have different IC_50_ values against DPP-IV. The distinction arises from the fact that diverse phytochemicals, such as glycosides, flavonoids and phenols, terpenoids, and stilbenoids, are located in different parts of various plants. Numerous bioactive peptides derived from various plant components are likewise highly efficient against DPP-IV activity (**Figure 3**).

### DPP-IV inhibitors in the leaves of plants

Many phytochemicals, including some that specifically target the DPP-IV enzyme, can be found in the leaves of plants. The leaves contain the most DPP-IV inhibitory compounds compared with other parts of the plant. To identify DPP-IV inhibitory compounds, the leaves of various plant orders, including Myrtales, Fabales, Sapindales, Gentianales, Urticales, Ericales, and Liliales, have been studied (**Figure 3**)—for example, the leaves of different plants from the order Myrtales, such as *Psidium guajava* (29), *Eucalyptus globulus*, *Eucalyptus citriodora*, and *Eucalyptus camaldulensis* (30), have IC_50_ values against DPP-IV activity of 380, 3.098, 6.138, and 3.99 μg ml^-1^, respectively. Similarly, the IC_50_ value against DPP-IV activity in the leaves of *Cicer arietinum*, *Pterocarpus marsupium*, and *Punica Granatum* belonging to order Fabales are 0.09 (31), 273.73 (29), and 0.19 μg ml^-1^ (32), respectively. *Aegle marmelos*, *Mangifera indica*, and *Melicope glabra*, which belong to the order Sapindales, have an IC_50_ value against DPP-IV activity of 790, 182.7, and 169.40 μg ml^-1^ (33–35), respectively. The IC_50_ value was 96.8 µM (36) and 773.22 μg ml^-1^ (16) in a few other plants such as *Apocynum cannabinum* and *Gymnema sylvestre* from the order Gentianales. Similarly, for several other plants such as *Morus alba* and *Pilea microphylla* (order Urticales), *Aloe vera* (order Liliales), *Camellia sinensis* (order: Ericales), and *Urena lobate* (order: Malvales), the recorded IC_50_ value against DPP-IV activity was 480 (37), 520.4, 2.716, 227, and 1,654.64 μg ml^-1^, respectively (37–39). These values show that the leaves of many plants have a very important role in inhibiting DPP-IV activity.

The leaves of *Fagopyrum esculentum* and *Rheum palmatum* (order: Chenopodiales) containing rutin and emodin were found to have IC_50_ values of 485 µM (27) and 5.76 µM (40), respectively. Fan et al. (2013) (41) documented that the leaves of *Camellia sinensis* from the order Ericales have gallic acid and epigallocatechin gallate having IC_50_ values of 4.65 and 10.21 µM, respectively. Against DPP-IV activity, the leaves of *Psoralea corylifolia* (order: Fabales) contain genistein, *Coffea arabica* (order: Gentinales) contains caffeic acid, and *Brassica oleracea* (order: Brassicales) contains luteolin with IC_50_ values of 0.48, 3.37, and 0.12 M, respectively (41). Similarly, Bower et al. (2014) (14) observed that the leaves of *Rosmarinus officinalis* and *Lippia graveolens* (order: Lamiales) have cirsimaritin with IC_50_ value of 0.43 and 10.9 µM, respectively, which can be used for inhibition of DPP-IV activity.

### DPP-IV inhibitors in plant seeds

The seeds of various plants belonging to different orders, such as *Castanospermum austral*, *Cicer arietinum*, *Trigonella foenum graceum* (order: Fabales), *Avena sativa*, *Hordeum vulgare* var. trifurcatum (order: Graminales), *Amaranthus hypochondriacus* (Order: Chenopodiales) *Eugenia jambolana* (order: Myrtales), *Fagopyrum esculentum* (Order: Polygonales), *Ferula assa-foetida* (order: Apiales), and *Prunus amygdalus* (order: Rosales), were documented to have IC_50_ concentration against DPP-IV activity of 13.96 (42), 0.09, 0.03 (31), 0.99, 1.83 (43), 1.1 (44), 278.94 (32), 1.98 (43), 24.5 (45), and 162.9 μg ml^-1^ (46), respectively. Kim et al. (2018) (47) also examined that the seeds of *Lens culinaris* (order: Fabales) have different compounds such as kaempferol-3-O-βgulcopyranosyl-(1→2)-βgalactopyranosyl-7-Oαrhamnopyranoside, kaempferol-3-O-βgulcopyranosyl-(1→2)-[αrhamnopyranosyl(1→6)]-βgalactopyranosyl-7-O-αrhamnopyranoside, robinin, and kaempferol with IC_50_ value against DPP-IV activity of 27.89, 36.52, 37.01, and 51.9 µM, respectively.

### DPP-IV inhibitors in other parts of plants

Several additional plant parts have been identified to have compounds with DPP-IV activity-inhibiting properties. The aerial parts of *Hedera nepalensis* (order: Apiales), *Fagonia cretica* (order: Zygophyllales), and *Desmodium gangeticum* (order: Fabales) have IC_50_ value against DPP-IV activity of 17.2 (19), 38.1 (19), and 255.5 μg ml^-1^, respectively. Similarly, the IC_50_ values for *Helichrysum arenarium* (order: Asterales) flowers, *Berberis aristate* (order: Ranunculales) bark, and *Anogeissus latifolia* (order: Myrtales) were reported as 41.2, 14.4, and 754 μg ml^-1^ (34, 48), respectively, for DPP-IV inhibition. The bulb of *Allium sativum* (Order: Asparagales) was likewise documented with an IC_50_ value of 70.9 μg ml^-1^ (49), while the fruits of *Schisandra chinensis* (Order: Austrobaileyales) and *Punica granatum* (Order: Fabales) were recorded with an IC_50_ value of about 10.8 μg ml^-1^ (31, 32) against DPP-IV activity.

Saleem et al. (2014) and Kalhotra et al. (2018) (19, 49) noticed that lupeol, present in the aerial parts of *Hedera nepalensis* (Order: Apiales), has an IC_50_ value of 31.6 µM, but malvidin, found in the aerial parts of *Anagallis monellin* (Order: Ericales), has an IC_50_ value of 1.41 µM against DPP-IV activity. The stems and roots of *Coptis chinensis* (Order: Ranunculales) contain berberine, which exhibits DPP-IV activity with an IC_50_ value of 14.4 μg ml^-1^ (13). The whole *Pilea microphylla* (Order: Rosales) plant contains the active ingredient isoquercetin, which has an IC_50_ value of 96.8 µM against DPP-IV activity (50). Lin et al. (2015) (28) identified hopeaphenol, vitisin A, and vitisin B in the stems and leaves of *Vitis thunbergii* that inhibited DPP-IV activity with IC_50_ values of 401, 90.75, and 15.3 µM, respectively. Similarly, Fan et al. (2013) (41) found apigenin in the stems and pods of *Acacia auriculiformis* (order: Fabales), which had an IC_50_ value of 0.14 µM, in the fruits of *Citrus aurantium*, *Citrus limon*, and *Citrus maxima* belonging to order Sapindales having hesperetin (with IC_50_ value of 0.28 µM), eriocitrin (IC_50_ value of 10.36 µM), and naringenin (IC_50_ value of 0.24 µM), respectively. They also recorded that the fruits of *Rubus fruticosus* belonging to order Rosales have cyanidin (IC_50_ value 1.41 µM) that acts as a DPP-IV inhibitor. The compound cyanidin-3-glucoside present in *Vaccinum corymbosum* (order: Ericales) has an IC_50_ value of 125.1 µM against DPP-IV activity (26).

### DPP-IV inhibitor peptides

It has been demonstrated that several bioactive peptide sequences found in a wide variety of plants are effective against DPP-IV activity. In *Phaseolus vulgaris*, Mojica et al. (2017) (18) identified peptide sequences such as KTYGL, KKSSG, GGGLHK, and CPGNK, each of which had an IC_50_ value of 0.03, 0.64, 0.61, and 0.87 mg DW ml^-1^, respectively. Similarly, Lammi et al. (2016) (17) reported that the IC_50_ value for the peptide AVPTGVA in *Glycine max* and LTFPGSAED in *Lupinus albus* was 106 and 228 µM, respectively. Wang et al. (2015) (43) determined that the peptide LQAFEPLR present in *Avena sativa* has an IC_50_ value of 103.5 µM. Harnedy et al. (2015) (15) found that ILAP and LLAP present in *Palmaria palmata* had IC_50_ values of 43.40 and 53.67 µM, respectively. The details about these plants are specified in Section 9.

## Pharmacovigilance status of DPP-IV inhibitors

With an ideology to work on the crux of the matter, DPP-IV to manage diabetes, orally active small molecules called DPP-IV inhibitors have already been introduced in the market since 2006 (51). From previous investigations, a therapeutic dosage of different authorized DPP-IV inhibitors led to a two- to threefold raise in endogenous GLP-1 concentration without any inherent hypoglycemia risk and a favorable safety profile (**Figure 2**). Synthetic authorized drugs in the market as DPP-IV inhibitors are known as gliptins such as sitagliptin, linagliptin, and saxagliptin, usually substantiated to be efficient competitive inhibitors. In order to address the crisis imparted by DPP-IV, sitagliptin (Merck) was the initial lead to embark on the journey towards the upregulation of GLUT-4 expressions in the skeletal muscles of spontaneously hypertensive rats by controlling the postprandial glucose concentration and glycated hemoglobin (52). With an appropriate drug administration, gliptins can prove their efficacy for 24 h (53). Moreover, from the pharmacovigilance studies done priorly, gliptins were able to curtail the risk of hypoglycemia and weight loss and additionally also had the potential to proliferate β-cell mass.

Variously targeted (GLP-1) and off-targeted substrates lead to DPP-IV inhibition, contributing to managing incretin activity and maintaining the basal blood glucose level (54). Lately, these oral hypoglycemic agents, because of their insulinotropic effect, have progressively replaced sulfonylurea as second-line therapy and are also endorsed in the guidelines in triple therapies along with metformin and SGLT-2 inhibitors (55–57). For attaining the benchmark among all the other synthetic drugs, structural backbones ranging from cyanopyrrolidines, triazopiperazine amides, and pyrrolidines highly influenced the pharmacodynamics of each commercialized gliptins (58). The pharmacodynamics of each commercially authorized gliptin has been summarized in **Table 1** (54, 59).

**Table 1:** Commercial dipeptidyl peptidase-IV inhibitors.

Attributes of marketed drugs	Sitagliptins	Vildagliptins	Saxagliptins	Alogliptins	Linagliptin
DPP-IV inhibition (%)	More than 80%–90%	More than 90%	75%–80%	More than 80%	More than 80%
Specificity for DPP-IV and type of inhibition	Very high, competitive, and dose-dependent inhibitor	High affinity (but not as sitagliptin) as compared with sitagliptin; it acts as a substrate blocker	Moderate affinity, selective, reversible, competitive inhibitor	High affinity, competitive, and dose-dependent inhibitor	High affinity, high specificity, and dose dependent inhibitor
HbA1C reduction (%)	0.6% only sitagliptin 0.89% with metformin	0.7%	0.45–0.65%	0.6% with sitagliptin 0.7% with vildagliptin	0.53%
Hypoglycemia risk	No risk detected	No risk detected	No risk detected	No risk detected	No risk detected
Mean half life	8–14 h	1.3–2.4 h	2.5 h	12.4–21.4 h	12 h
Bioavailability	±87%	±85%	±67%	100%	±30%
Metabolism/elimination	Primary route: kidneys (only 16%) About 74% has been accounted for as parental drug	Primary route: kidneys About 85.04% fraction of the drug is absorbed and recovered in urine, wherein 27.14% (22.60 % recovered in urine and 4.54% recovered in feces) is unchanged parental drug and 57.90% is after hydrolysis	Primary route: kidneys and liver Liver: metabolizes the drug by cytochrome P450 enzymes and forming an active metabolite Kidney: as renal circulation 22.1% as an unchanged parent compound and 44.1% as a metabolite	About 60% to 80% of the administered dose tends to be unchanged in the urine after 24 to 72 h and 10%–20% of the dose is hepatically metabolized by cytochrome enzymes Primary excretion: kidneys (76%) Secondary: fecal (13%) Two minor metabolites explored were N-demethylated alogliptin (inhibitor of DPP-IV) and N-acetylated alogliptin	About 70%–80% of the administered drug is bound to plasma proteins Primary excretion: bile and gut Secondary excretion: kidneys
Year of approval	2006	2007	2009	2013	2011
Brand name	Januvia	Galvus	Onglyza	Nesina	Tradjenta

Data are partially retrieved from the approved list of DPP inhibitors by the US Food and Drug Administration (FDA) (60). FDA has underlined the adverse effects of DPP-IV inhibitor drugs such as Januvia, Janumet, Janumet XR, Onglyza, Kombiglyze XR, Tradjenta, Glyxambi, Jentadueto, Nesina, Kazano, and Oseni with active compounds sitagliptin, sitagliptin and metformin, sitagliptin and metformin extended release, saxagliptin, saxagliptin and metformin extended release, linagliptin, linagliptin and empagliflozin, linagliptin and metformin, alogliptin, alogliptin and metformin andalogliptin, and pioglitazone, respectively (60).

## Shortcomings associated with DPP-IV inhibitors

The previous studies revealed the most common adverse effects in patients, including infections in the respiratory passage, nasopharyngitis, headache, and bladder infections associated with sitagliptin and saxagliptin, respectively (61, 62). Moreover, cases of hypoglycemia were also encountered with the synergistic intake of sitagliptin and saxagliptin. From analyzing the pharmacovigilance report and data supported by FDA, it was proclaimed that, on intake of DPP-IV inhibitors, several patients were diagnosed with hypersensitivity and joint pain issues, and in some cases, these drugs have proven to be fatal for patients with a history of pancreatitis (7, 56, 61, 63).

On a wider scale, strong aversions were laid against incretin-based therapy regarding its cost-effectiveness and the increasing risk of morbidity in patients with cardiovascular issues (64). With an ideology to surpass the discredits, investigators have inclined toward plant origin to discover innovative therapies to combat glycemia and the emergence of severe morbidities associated with the same.

## Pharmacognosy: A strategy to alleviate the glycemic load

Since antiquity, many traditional practitioners across the globe have assertively exploited natural flora, which has proven superlatively therapeutic over synthetic agents to regulate several pathological conditions. Botanical leads, being a repository of bioactive compounds, fostered the strategic role of pharmacognosy (65). Metformin, a commercially effective anti-diabetic medicine, was recently identified in the field of drug discovery by adapting the concept of pharmacognosy and establishing the foundation for formulating herbal products to inhibit DPP-IV and regulate incretin activity (7, 66, 67).

## Potential benefits of DPP-IV inhibitors with antioxidant properties in treating diabetes

Oxidative stress (OS) has been linked to the onset and progression of diabetes and its many consequences resulting from insulin insufficiency or insulin resistance (68). Several clinical studies have demonstrated that T2DM reduces antioxidant status and free radical scavenging activity due to the decreased activities of superoxide dismutase (SOD), catalase (CAT), and glutathione peroxidase (GPx) and ascorbate and vitamin E levels, increasing the likelihood of diabetic patients acquiring chronic OS (68). Several different molecular mechanisms such as protein modifications, including oxidant-induced modifications in phosphorylation state, alterations in gene regulation including transcriptomic and transcriptional modifications with the affected proteins being either direct insulin signaling molecules or oxidant-sensitive signaling pathways that interfere with the insulin signaling cascade, have been postulated to contribute to reactive oxygen species (ROS)-induced insulin resistance (69). Excessive levels of ROS inhibit insulin gene expression and insulin production as well as damage islet tissue. Anti-diabetes effects can be enhanced using plant-based DPP-IV inhibitors, such as alkaloids, phenolic acid, flavonoids, quercetin, and coumarin, possessing antioxidant characteristics (70, 71).

**Figure 4:**
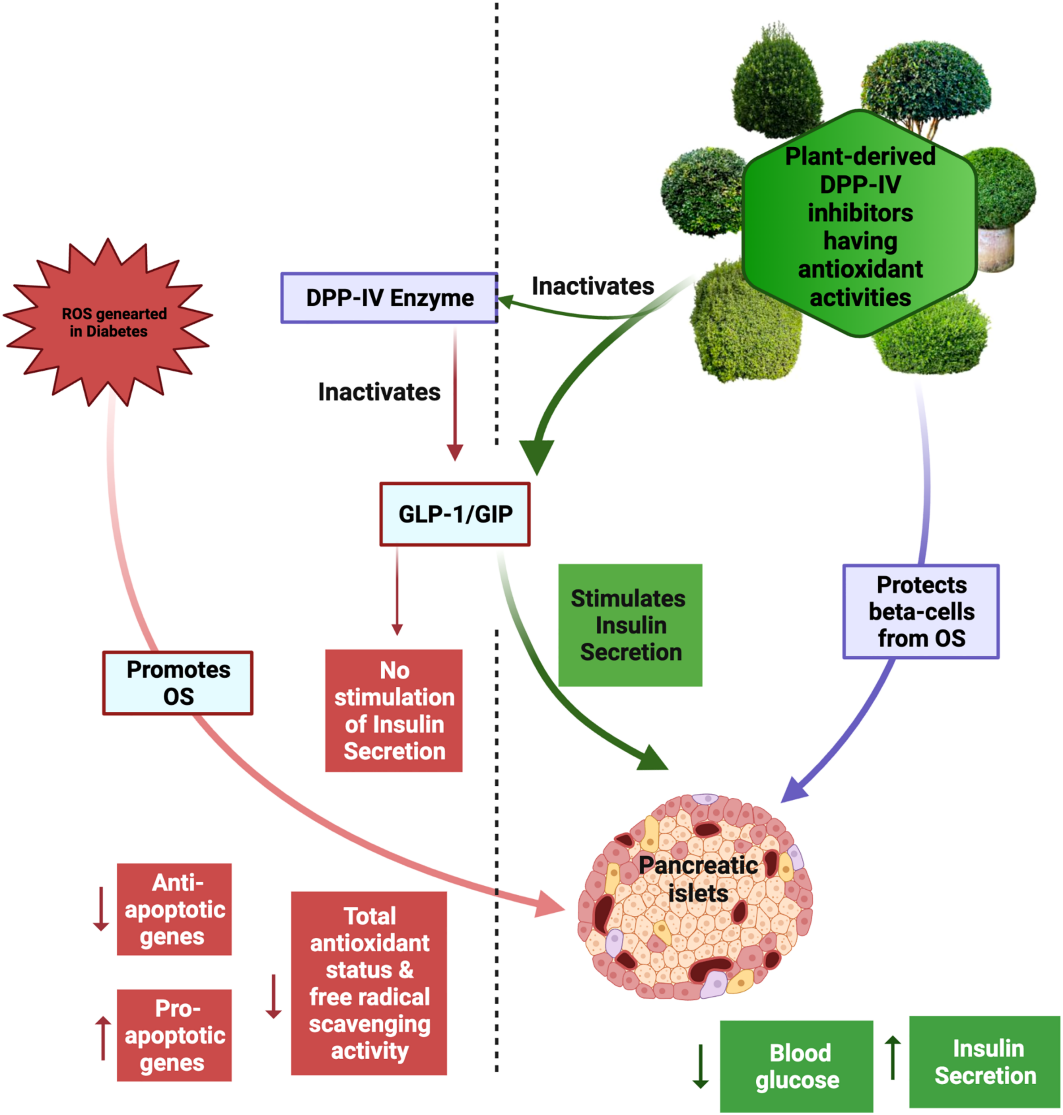
Role of dipeptidyl peptidase inhibitors with antioxidant properties in protecting pancreatic β-cells and treating diabetes. GLP-1, glucagon-like peptide-1; GIP, glucose-dependent intestinal polypeptide; OS, oxidative stress.

Antioxidants derived from plants, such as kinsenosides and flavonoids, exhibit anti-diabetic and antioxidant properties and assist in maintaining the function of pancreatic β-cells *in vivo* (71, 72). The majority of studies examining the role of plant-derived antioxidants in protecting β-cells focus on flavonoids. The administration of flavonoids to diabetic animals boosts the antioxidant potential of β-cells by increasing both enzymatic and non-enzymatic antioxidants, consequently limiting ROS generation and lipid peroxidation in β-cells and protecting them against autophagy, apoptosis, or necroptosis (73, 74). Diabetes is associated with an increase in the expression of pro-apoptotic genes (*e*.*g*., caspases) and a decrease in the expression of anti-apoptotic genes (*e*.g., Bcl-2 proteins) in β-cells. Flavonoids have been proven to protect β-cell viability by limiting these gene expression changes (**Figure 4**) (71, 75).

Flavonoids, which include flavanols, flavonols, flavones, flavanones, isoflavones, and anthocyanins, also possess DPP-4 inhibitory activity—for instance, the flavonoid-rich fraction of *Pilea microphylla* displayed antidiabetic efficacy in rats with diabetes generated by a high-fat diet and streptozotocin by lowering DPP-IV. This was accomplished while simultaneously increasing the endogenous antioxidant status in the liver of mice (38). Similarly, supplements derived from citrus bioflavonoids suppress the DPP-4 enzyme and also have higher free radical scavenging capabilities (27, 76). It has also been reported that an ethanolic extract of the leaves of *Psidium guajava* contains seven major flavonol-glycosides, all of which inhibit DPP-IV in a dose-dependent manner (29). Additionally, it was demonstrated through an *in vitro* bioassay that three flavonol glycosides extracted from the seeds of *Lens culinaris* possessed DPP-IV inhibitory activity in a concentration-dependent manner (47). Antioxidants derived from plants with antidiabetic qualities, such as acting as DPP-4 inhibitors, are regarded to be the most effective strategy for keeping a normal β-cell physiology and treating diabetes (77). The following sections address plants that have DPP-4 inhibitor actions, either with or without known antioxidant properties.

## *In vitro* and *in vivo* studies of DPP-IV inhibitors derived from plants

The effectiveness of the DPP-IV inhibition offered by a variety of tropical plants is being evaluated. In addition, in prior studies, a wide variety of organic solvents, plant parts, and extraction procedures were employed to isolate different bioactive components. The percentage yield of the extracts and the number of bioactive compounds are profoundly influenced by the polarity of the organic solvents (39, 78).

### *Urena lobata*: Pharmacokinetic difference between *in vitro* and *in vivo* inhibition efficacy against DPP-IV

Recent studies on the root and the aqueous leaf extract of *U. lobata* reported its anti-diabetic competency in streptozotocin-induced diabetic rats, in compliance with the fact that the polarity of the organic solvent affects the solubility of bioactive compounds and its antioxidant property, which is supported by *in vitro* and *in vivo* generated results (79, 80). As a result of the research performed *in vitro*, the findings of the alcoholic extract portrayed its inhibition efficacy by expressing an IC_50_ value of 1,654.64 µg/ml. This value demonstrated that the alcoholic extract was four times more effective than the aqueous extract, which had an IC_50_ value of 6,489.88 µg/ml. However, the *in vitro* inhibitory activity of standard vildagliptin was superior to both extracts (IC_50_ = 57.44 µg/ml) (39, 78). *In vivo*, however, pharmacokinetic indices indicated that the aqueous extract was more effective than the alcoholic extract because water positively influences the absorption of active compounds in the serum by synergistically forming a complex and enhancing the inhibition efficacy against DPP-IV as well as the bioavailability of GLP-1 in the serum (81). Because the extracts contain sterols, which are known to alter the conformational structure of active compounds and subsequently reduce the bioactivity within the gastrointestinal (GI) tract, the alcoholic extract displays a lower level of *in vivo* inhibitory activity (39, 82, 83).

### *Pueraria tuberose: In vitro and in vivo* inhibition efficacy against DPP-IV

From the *in vivo* studies undertaken previously using *P. tuberose* methanolic root extract (PTME) as a potential herbal treatment on the liver homogenates of alloxan-induced diabetic rats, it was reported that PTME possessed an abundance of flavonoids and significantly reduced DPP-IV activity in a time-dependent manner from 16 DPP-IV activity in a time-dependent manner fromto 4 μM/unit as compared with alloxan control (17.5 μM/unit) within a period of 40 days, which proved that PTME was a promising candidate to cure DPP-IV-induced liver disorders (84). From another investigation done, the aqueous extract was found to be potent enough to upregulate antioxidant SOD levels and downregulate DPP-IV mRNA expression, which apparently led to a concomitant reduction in stress, changes in the structural complexity of villi, increase of the intestinal patch, subsequent increase of the rate of nutrient absorption, and augmentation of GLP-1 and GIP secretion by causing an enhanced intestinotrophic effect (85). Besides this, *in vitro* results also supported the data as a potential DPP-IV inhibitor by exhibiting a considerable IC_50_ value of 17.4 mg/ml. In addition, when *in vivo* studies were undertaken on glucose-fed rats administered with the aqueous extract, the plasma glucose concentration during 60 min was reduced by 27.68%, and plasma DPP-IV activity was reduced by approximately onefold as compared with untreated rats. Additionally, a sharp rise of 1.2-fold was also observed in the plasma GLP-1 concentration compared with the untreated control (84).

### *Withania coagulans: In vitro* inhibition efficacy against DPP-IV

As one of the prospective candidates, *W. coagulans* was chosen, and its effectiveness was checked using root, leaf, and fruits with 100 and 80% methanol as an organic solvent. Among all the plant parts, a root extract of the mature plant with 100% methanol exerted the maximum 50% inhibition efficacy against DPP-IV at a concentration of 8.76 µg/ml, whereas 80% methanol expressed its 50% inhibition at a concentration of 21.03 µg/ml. However, both extracts showed lesser efficacy than diprotin, the standard drug expressing its 50% inhibition efficacy at a concentration of 4.13 µg/ml (86).

### *Commiphora mukul*: Choice of extraction method highly influences the extract yield

Recent findings have unveiled that the alcoholic extract of *C. mukul* gum resin with an abundance of antioxidants significantly proclaimed its antihyperglycemic activity by modulating the key glucose-metabolizing enzymes of the liver and kidney and further promoting normal blood glucose homeostasis (87–89).

With an ideology to get the maximum yield, optimization of procedure was an essential need which was undertaken by microwave-assisted extraction (MAE) method and conventional Soxhlet extraction (CSE) method resulting from giving a yield of 2.5%–3% and 2%, respectively, by using ethyl acetate as the solvent of interest (90). MAE proved to show a better yield than the Soxhlet extraction method because it was found to be less laborious, required less solvent, with improved quality of extract, and more economical (91). Moreover, by taking Hydro alcohol (HA) extract into practice for obtaining *in vitro* results, the maximum inhibitory activity imparted by C. *mukul* was 92%, with an IC_50_ value of 17 µM.

### *Ferrula asafetida*: *In vitro* inhibition efficacy against DPP-IV

When experiments were undertaken by choosing *F. asafoetida* to assess DPP-IV inhibition activity using methanol, ethanol, water, and methanol–ethanol as organic solvents, it was observed that, among all the fractions, ethanol and ethanol–methanol fractions showed a maximum inhibitory effect with 24% and 22%, respectively (45).

### *Desmodium gangeticum*: *In vitro* inhibition efficacy against DPP-IV

By choosing *Desmodium gangeticum* as one of the potential DPP-IV inhibitors, it was noted that the aqueous extract exhibited 73.21 % inhibition at 1,000 µg/ml concentration with an IC_50_ value of 255.5 µg/ml, which was inferior concerning its efficacy when compared with the standard drug diprotin expressing IC_50_ =10 µg/ml by 78.3% (92).

### *Moringa oleifera*: *In vitro* DPP-IV inhibitory activity

According to the data, the leaf extract of *Moringa oleifera* was considered one of the potential candidates with significant antihyperglycemic activity, particularly in the ethanol and ethyl acetate extracts (93). Recent findings suggested that the extracts successfully reduced the blood glucose levels, increased the insulin levels, decreased the inflammatory cytokines and HOMA-R values, and improved the PPAR gamma levels. Nevertheless, the extract failed to prove its inhibition efficacy against DPP-IV when a set of *in vivo* experiments was performed (94). However, by undergoing ADMET analysis to evaluate the pharmacokinetic indices, myriads of different compounds from the extract were screened. Seven out of all could make it through *in silico* analysis by undergoing molecular docking, and just one compound with a specific conformation that exhibited the highest inhibitory activity with maximum binding energy was selected. Its *in vitro* evaluation was also undertaken, where it responded with an IC_50_ value of 798 nM (95).

### *Morus alba*: Sample pre-treatment influences the *in vitro* DPP-IV inhibitory activity

Recent data has also reported *Morus alba* (also called white mulberry) as a potential DPP-IV inhibitor. MAE was used to obtain the extract from dried stem bark using ethanol with and without hydrolyzed acid. In addition, it was observed that the proportion of bioactive compounds in the *Morus alba* ethanolic extract without acid hydrolysis was only 0.04% but that the percentage of these compounds increased to 0.16% when the extraction was carried out with acid hydrolysis (96). Furthermore, previous research findings suggested that the compounds from *M. alba* root bark and fruits have also been reported to have anti-diabetic effects on STZ-induced mice by stimulating insulin secretion but lack the bioactive compounds which could exert DPP-IV Inhibition (97–100). The experiments were conducted to check the efficacy of stem bark as a DPP-IV inhibitor, wherein it showed a considerable inhibitory activity of 23%, which was 0.33 times the inhibitory activity of the standard drug sitagliptin (96).

### *Pterocarpus marsupium*: *In vitro* and *in vivo* inhibition against DPP-IV

*Pterocarpus marsupium* was selected to treat hypoglycemia by utilizing various parts of the plant, and the DPP-IV inhibitor activity of the plant was investigated in both *in vitro* and *in vivo* conditions. Based on the findings, it was possible to determine an IC_50_ value of 273 µg/ml. In addition, the experimental conditions impacted the inhibitory half-life of the enzyme, which was 462.3 min, whereas the *in vivo* studies also reported that the extract could successfully increase the GLP-1 levels compared with the control group; the highest peak for GLP-1 was detected within 2 h (32).

### *Curcligo latifolia*: *In vitro* inhibition against DPP-IV

As one of the potential options for combating hyperglycemia and hyperinsulinemia, the Hypoxidaceae family member *C. latifolia* has been selected. The root and the fruit extracts were prepared using the subcritical water extraction method. Both extracts expressed inhibitory potential against DPP-IV, and from the results, it was portrayed that the root extract (66.15%) exhibited better inhibition against DPP-IV than the fruit extract (42.79%).

### *Melicope latifolia*: *In vitro* inhibition against DPP-IV

In the latest studies, the anti-diabetic and antioxidant potential of *M. latifolia* has been demonstrated, and the bark extract obtained from the plant has been investigated using various solvents. Methanol showed the highest yield out of all the evaluated extracts. Moreover, the *in vitro* results depicted that chloroform extract depicted the maximum inhibitory strength against DPP-IV (IC_50_ value = 221.58 µg/ml), whereas the hexane extract expressed the minimum inhibitory potential.

## Phytochemical screening and *in silico* analysis

Poor pharmacokinetic characteristics are a common reason many drugs never make it to the market (101) (**Table 2**). Therefore, it is crucial to develop lead compounds that are easily absorbed orally, efficiently delivered to the site of action, not readily converted into harmful metabolic products *en route* to the site of action, and rapidly excreted from the body. The acronym ADMET is commonly used to describe the aforementioned characteristics (absorption, distribution, metabolism, excretion, and toxicity). Although Lipinski's “rule of five" (106) is an early version of an integrated approach, its parameters should be used for guidance rather than as strict cutoffs. This mnemonic predicts that poor absorption or permeation is more likely when there are more than five hydrogen-bond donors and more than 10 hydrogen-bond acceptors, the molecular weight is greater than 500, and the calculated partition coefficient (logP) is greater than five. The same holds for the methods used to deal with problems caused by specific ADMET attributes (107).

**Table 2:** Inhibitory role of different tropical plant extracts against dipeptidyl peptidase-IV enzyme.

Plant	Family	Part used in the investigation	Organic solvent	IC_50_ value/percentage inhibition	Extraction method	References
*Urena lobata*	*Malvaceae*	Root	Water Ethanol	6,489.88 µg/ml 1,654.64 µg/ml	Decoction	(78)
*Pueraria tuberose*	*Fabaceae*	Root	Water Methanol	17.4 mg/ml Activity is reduced in a time-dependent manner	Decoction Continuous soxhlet	(84, 102)
*Withania coagulans*	*Solanacea*	Root	Methanol (80%, 100%) Ethanol	8.76 µg/ml 21.03 µg/ml	Hot percolation	(86, 103)
*Ferrula asafetida*	*Umbelliferae*	Seed	Methanol Ethanol Water Methanol–ethanol	24%	Percolation	(45)
*Desmodium gangeticum*	*Leguminosae*	Aerial parts	Water	255.5 µg/ml	Maceration	(92)
*Moringa oleifera*	*Moringaceae*	Leaf	Ethanol Ethyl acetate Hexane Aqueous	798 nm	Percolation	(93, 94)
*Morus alba*	*Moraceae*	Stem bark	Methanol	23%	MAE	(96)
*Pterocarpus marsupium*	*Leguminosae*	Root Stem Leaf	Petroleum ether Methanol Ethyl acetate Chloroform	273.73 µg/ml	Soxhlet and maceration	(32)
*Curcligo latifolia*	*Hypoxidaceae*	Root Fruit	Methanol Ethanol Acetonitrile	7.02% to 66.15% 2.69% to 42.79% (increased with respect to concentration dependent)	Subcritical water extraction	(104)
*Melicope latifolia*	*Rutaceae*	Bark	Methanol Chloroform Hexane	990.21 µg/ml 221.58 µg/ml 5,872.03 µg/ml	Maceration	(105)

**Figure 5:**
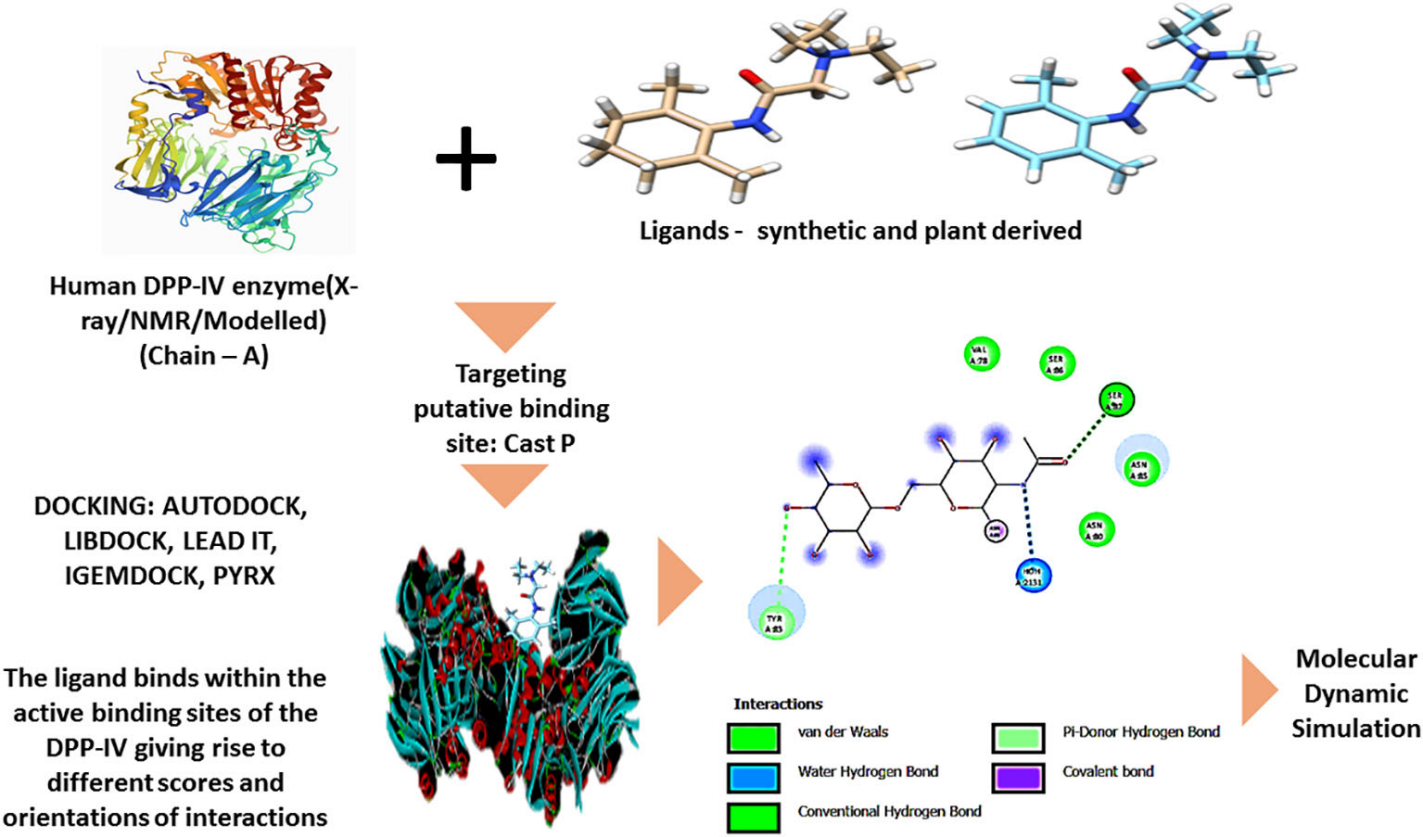
General mechanism of molecular docking. With a view to check the inhibition efficacy of different phytocompounds against the dipeptidyl peptidase (DPP)-IV putative binding site, *in silico* analysis can be performed by exploring various software, and in accordance with the same, different binding scores and orientations can be known, which are further projected towards molecular dynamics simulation. The ribbon structure of the human DPP-IV and the molecular structure of the ligand were downloaded from the protein data bank of PubMed and PubChem, respectively. The molecular interaction figure is recited after Kim et al. (2018) (47) under creative common license attribution.

A broad variety of bioactive compounds have been identified through the use of various plants and various ways of extraction. In addition, over the course of time, certain novel bioactive compounds have been explored for their potential as DPP-IV inhibitors through the use of *in silico* analysis (**Figure 5**). With the aid of advanced computational biotechnological studies, scientists can foretell the potential drug candidates in the pharmaceutical industry. New ligands are needed for the targets of known structure, wherein the potential compounds are screened for their adequate efficacy against a target of interest, and also the therapeutic dosage approval is possible by analyzing the ADMET properties to be able to abide by the Lipinski rule and investigate the drug likeliness of the compound (108, 109). The score of the free binding energy is directly proportional to the binding affinity. The binding energy with a lower value reinforced the synthesis of a strong binding molecule by exhibiting potential biological activity and binding orientation field (109, 110).

By generating an ensemble of ligand conformers that are subsequently firmly docked into the binding site, fast rigid exhaustive docking (FRED; part of the OpenEye Scientific Software) enables ligand conformational flexibility. Fast computations are possible with FRED, which is useful when screening libraries containing hundreds of thousands of molecules (111). A FRED docking study indicated that inhibition of DPP-IV is one of the mechanisms by which berberine exerts its hypoglycemic impact (13). Amini et al. (2016) performed molecular docking experiments and modeled the DPP-IV inhibitory activities of a variety of new aminomethyl-piperidones using a quantitative structure–activity relationship (QSAR) technique (112). This paper demonstrates the utility of a hybrid docking-QSAR approach to the study of ligand–protein interactions. In another study, three of the sugars found in fructooligosaccharides (*i*.*e*., 1-kestose, nystose, and 1-β-fructofuranosyl nystose) are powerful inhibitors of dipeptidyl peptidase-IV as determined by molecular docking with Gold and Glide software (113). Furthermore, with the results of the isobologram and combination index analysis, the synergistic effects were also evaluated showing nystose and panose combinations having the greatest synergistic effects (114). In a recent report, the interaction of flavonoids with DPP-IV has been studied elaborately (115). Among a panel of 70 structurally different flavonoids that were tested for their inhibitory properties, myricetin, hyperoside, narcissoside, cyanidin 3-O-glucoside, and isoliquiritigenin exhibited concentration-dependent stronger inhibitory effects against DPP-4. Furthermore, fluorescence quenching studies revealed that these five flavonoid molecules could efficiently suppress the intrinsic fluorescence of DPP-4 by forming an unstable complex through spontaneous binding. While myricetin's complex with DPP-4 was mostly stabilized by hydrogen bonds and van der Waals forces, electrostatic forces may play a significant role in maintaining the complexes of the other four flavonoids with DPP-4. AutoDock 4.2 software was used to perform the molecular docking simulation, which further confirmed the binding interactions between DPP-4 and the five flavonoids chosen. The binding sites of hyperoside, narcissoside, cyaniding 3-O-glucoside, and isoliquiritigenin were found within the active site cavity of DPP-4, while myricetin's binding site was found in a minor cavity close to the active pockets (115).

Most of the flavonoids and terpenoids in the herbs have potency as antioxidants (116), antiseptics, and anti-inflammatory, and sterols are known for their anti-inflammatory property, but the pharmacological influence of alkaloids is difficult to predict because of the presence of many biological activities. Anti-diabetic herbs have many active compounds which could either work synergistically or antagonistically (117).

To reduce the pathogenesis of diabetes and its complications, flavonoids and polyphenolics have played an extremely significant role by potentiating GLUT-4 (**Figure 1**) isomer expression and uptake (118). Flavonoids portray their characteristic antidiabetic potency by promoting carbohydrate digestion, insulin signaling, secretion, glucose uptake, and adipose deposition (119). From the previous findings, a strong notion was developed that the phytocompounds have a potency to regulate GLUT-4 translocation through insulin signaling pathways, namely, the PI3K-Akt pathway and AMPK-dependent pathway (120). Moreover, even the phenols trigger the secretion of GLP-1 from the L cells of the large intestine, causing the elevation of the half-life of GLP-1 and the inhibition of DPP-IV enzymatic activity (121). Synergistically, many phytocompounds also showcased their antioxidant and anti-inflammatory effect by suppressing oxidation and inflammatory signaling pathways and further preventing the risk of developing diabetes-related complications.

The flavonoids obtained from the water and ethanolic leaf extract of *U. lobata* were chrysoeriol and gossypetin. Chrysoeriol is a flavone exhibiting anti-inflammatory properties, found to be soluble in water and alkali solution, and after undergoing liquid chromatography (LC)–mass spectrometry (MS), this bioactive compound was found to be present in both the ethanolic and aqueous leaf extracts, which were further explored by undergoing molecular docking to check its inhibition activity as a ligand against the enzyme DPP-IV, and its efficacy was proven by portraying the binding energy of -4.6 kcal/mol (78). Besides this, gossypetin was the other prominent flavone identified in the aqueous and ethanolic leaf extracts of *U. lobata* by undergoing LC–MS screening, and the inhibition activity of this bioactive compound was investigated by calculating the free binding energy, which was -5.20 kcal/mol (78).

Two of the most potent flavonoids, puererone and robinin, were obtained by undergoing HPLC–MS analysis from the aqueous root extract of *P. tuberose*, which was exploited through computational studies to investigate their potency as DPP-IV inhibitors. Both flavones identified were competitive inhibitors of DPP-IV as they docked directly into all the three putative active sites of DPP-IV by exhibiting direct interaction, hydrogen bonding, and pi–pi interaction to substantiate the strong binding affinity and inhibition activity towards DPP-IV by possessing free binding energies of 7.543 and 7.376 kcal/mol, respectively, which further validated the efficacy of PTWE (84).

To select a potent phytochemical for the development of a plant-based DPP-IV inhibitor, the fruit extract of *W. coagulans* was screened and subjected to computational analysis, and from the analysis, hydrogen bonding was analyzed for various bioactive compounds against the target enzyme. In accordance with the same, even the binding energies of the competent compounds were detected to range from -7.2 to -9.8 kcal/mol, further inhibiting the target enzyme irreversibly. Among all the phytocompounds screened, sitoindoside IX showed the maximum inhibitory activity (-9.8 kcal/mol), which was even higher than the standard drug sitagliptin (103).

To analyze the DPP-IV inhibitory activity of *Ferrula asafoetida* methanolic seed extract, 12 different bioactive compounds were isolated and characterized by undergoing gas chromatography (GC)–MS analysis; they were further screened to examine their inhibitory role by going through docking studies using AUTODOCK software. Among the 12 different bioactive compounds, four bioactive compounds—namely, hexadecanoic acid, methyl tetradecanoate, ethoxy-disilane, and 9,12-octadecadienoic acid—were found to be potent enough and can be used for *in vivo* studies (122).

Among different plants, even the seeds of *Moringa oleifera* could show its efficacy as a DPP-IV inhibitor when being addressed to different docking programs. This resulted in generating 23 different competent compounds as ligands to develop compatible configurations. This investigation revealed that seven out of 23 candidate compounds had higher LibDock scores than the standard vildagliptin. These seven were further subjected to CDOCKER program, and three of them showed the best potency as DPP-IV inhibitors by possessing binding energy better than vildagliptin. Among the three, compound 1, a unique urethane O-ethyl-4-[L-rhamnosyloxy) benzyl] carbamate, possessed the highest binding energy (-84.99 kcal/mol) (95).

Five different bioactive compounds were detected from the methanolic leaf extract of *Gynura bicolor*, which were characterized using HPLC and LC–MS analysis and subjected to docking to identify the compounds with strong binding efficacy. Among the evaluated bioactive compounds, 3-caffeoyl quinic acid showed the highest binding energy (-29.07 kJ/mol), which was higher than sitagliptin (-25.04 kJ/mol), linagliptin (-27.57 kJ/mol), and saxagliptin (-23.45 kJ/mol) and subsequently proved to be a promising inhibitor of the enzyme to regulate incretin activity (123).

By evaluating the *in vitro* activity of *Curcligo latifolia*, fractionation was performed, and five different bioactive compounds were obtained, which were then analyzed for their drug-like properties; phlorizin exhibited the highest binding energy (-10.9 kcal/mol), which was higher than that of the reference drug sitagliptin (-8.6 kcal/mol) (104), whereas the bioactive compounds of *Melicope latifolia* were isolated from chloroform extract by undergoing LC–MS. It was observed that, among the four different compounds, p-coumarate featured the maximum binding efficacy towards the target molecule (-5.7 kcal/mol) (105).

## Consortium of plant extracts—Polyherbal formulation

It has been discovered that several mechanisms in the treatment of diabetes effectively control the progress as well as alter the deteriorating condition of patients. Conventional medicines have been discouraged due to their adverse effects and withdrawal symptoms. In lieu of getting diabetes control, several herbal mixtures have been estimated and analyzed for treating diabetic patients. A traditional therapeutic herbal strategy has been practiced for the past many years. Furthermore, this strategy also confers its maximum effectiveness due to the synergistic action of several herbs to achieve better potency as compared with that of an individual herb, and this gave rise to the mechanistic concept of polyherbalism or polypharmacy, which could be looked at as one of the comprehensive approaches to address diabetes and its emerging complications (85, 124–126).

Moreover, nanoformulations could be used to address the limitations of herbal medicinal products, such as low stability and restricted absorption, which hinder their development as medicinal agents (127). Extracts from plants and isolated phytochemicals have been nanoformulated in a variety of ways to improve their therapeutic efficacy as nanoformulations and are proven to possess superior characteristics compared with the respective plant extracts or isolated phytochemicals. Prior research on peptides obtained from oat globulin revealed potent DPP4-inhibiting activity (128). Solid lipid nanoparticle-embedded oat globulin peptides were reported by Su et al. (2020) as stable and being able to maintain their capacity to inhibit DPP4, while non-embedded peptides suffered secondary hydrolysis by proteases in gastrointestinal fluids and lost their inhibitory effects (129). In a glucose-induced diabetic zebrafish model, *Eysenhardtia polystachya* (EP)-loaded silver nanoparticles (AgNPs) were likewise reported to boost pancreatic β-cell survival and insulin secretion-enhanced hyperglycemia. In addition, EP-AgNPs restored insulin production in insulinoma cell line (INS-1; an established model for studying the function of pancreatic islet β-cells) cells induced by H_2_O_2_, indicating that this may be due to cytoprotection against oxidative damage (130). Bark methanol/water extract from EP contains bioactive components including chalcones, flavonoids, and dihydrochalcones, which play a determining role in the phytofabrication of AgNPs (130).

Compared to the control Chang liver cells, glucose absorption was enhanced by the *Leonotis leonurus* extract-nanostructured lipid carriers (NLCs)  (131). Under hyperglycemic conditions, INS-1 cells exposed to the NLCs released more insulin than the control untreated cells and the unformulated-extract-treated cells  (131). A rise in the ATP/ADP ratio results from glucose metabolism in β-cells, which triggers insulin release. Increased intracellular Ca^2+^ stimulates insulin secretion by closing ATP-sensitive K^+^-channels, depolarizing the cell membrane, and activating voltage-gated Ca^2+^-channels (132). It has been demonstrated that a component derived from the *Leonotis sibiricus* plant can boost insulin production in INS-1E cells while simultaneously promoting cell proliferation (133). It is possible that the rise in insulin release caused by the extract NLC is attributable to the formulation's small particle size and high bioavailability (134), both of which lead to efficient cellular absorption, which is necessary for the effect to be achieved.

Paul et al. (2014) described an α-eleostearic acid (ESA)-rich nanoemulsion (NE) formulation and its anti-diabetic and antioxidative effects (135). Bitter gourd seed oil (BGO) contains 30%–50% ESA (136), whose antioxidative activity in scavenging ROS has been described (137). In plasma and liver homogenate fractions, the antioxidative defense system of rats with alloxan-induced T2DM was considerably impaired compared with the normal control group. Evidently, the administration of synthesized BGO-NE alleviated the oxidative stress state by activating the antioxidative defense mechanism (as demonstrated by an improvement in GPx, SOD, and CAT activities) (135).

## Clinical status of phytochemicals

For decades now, it has been established that endothelial dysfunction and, ultimately, atherosclerosis are directly linked to the increase in oxidative stress that occurs during the progression of diabetic hyperglycemia. According to prior research, plasma antioxidants, including tocopherol, carotene, lutein, lycopene, retinol, and ascorbic acid, decline significantly in diabetes (138). Many phytochemicals are utilized to treat various diseases, and they play a crucial role in the fight against free radicals by targeting myriads of enzymes in several metabolic pathways.

Recent studies show that quercetin and resveratrol are used to increase insulin sensitivity, whereas beta-glucans and basic acids have been clinically used to stimulate glycogen synthesis and gluconeogenesis. Scirupsin B and myricetin are clinically proven for efficacy by targeting amylase from the salivary gland. Curcumin and turmerin have been recorded to inhibit glucosidase in the small intestine. Additionally, chicoric acid and lupanine acids are administered for insulin secretion from β-cells. Even berberine and pectin imparted their efficacy for reducing fasting blood glucose, postprandial blood glucose, and hemoglobin A1c (HbA1c) (139). The previous studies also showed that the seed extract of *Castanospermum australe* possesses very strong DPP-IV inhibitory alkaloids, including castanospermine, 7-deoxy-6-epi-castanospermine, and australine, and these are depicted as their efficacy in the management of hyperglycemia in rats (42). Recent studies have also been undertaken with garlic bulb, which exhibited a substantial DPP-IV inhibition efficacy (IC50 = 70.88 μg/ml), and this was because of the presence of caffeic acid 3-glucoside, malonylgenistin, and calenduloside E which have very high binding energy **Table 3** (49). The recent *in silico* studies performed on the plant *Amberboa ramose* determined that the compound 5-hydroxy-7,8 dimethoxyflavone can be considered a promising candidate for serving well for all the purposes of drug likeliness by following the Lipinski’s rule (140). The phytochemicals extracted from *Commiphora mukul* are guggulsterone E, guggulsterone Z, and glucogallin, and these compounds have potent DPP-IV inhibitor activity compared with vildagliptin (standard plant drug). Additionally, phytochemicals like pzrogallol, beta-glucogallin, and gallic acid purified from the plant *Phyllanthus emblica* possess considerable inhibitory activity against the DPP-IV target compound (141).

**Table 3:** Different bioactive compounds and their respective binding energy value.

Plant	Phytochemicals	Bioactive compounds	Binding energy (kcal/mol)	References
*Urena lobata*	Flavonoids and flavones Sterols Glucoside	Gossypetin	-5.20	(78)
		Chrysoeriol	-4.66	
		Stigmasterol	-7.42	
		β-Sitosterol	-6.59	
		Mangiferin	-7.66	
*Pueraria tuberose*	Flavonoids	Robinin	7.543	(84)
		Puererone	7.376	
		Anhydrotuberosin	7.149	
		Daidzin	7.042	
		Tuberosin	6.965	
*Withania coagulans*	Sterols	Withanolide-D	-9.2	(103)
		Withanone	-7.9	
		Sitoindoside	-9.8	
		Withaferin	-8.1	
		Withacoagulin H	-8.9	
		Withanolide E	-7.6	
		Withangulatin A	-8.8	
*Ferrula asafetida*	Sterols	Hexadecanoic acid	-3.0	(122)
		Methyl tetra decanoate	-2.8	
		Ethoxydi (tert-butyl) silane	-2.4	
		9,12-Octadecadienoic acid	-2.7	
*Moringa oleifera*	Sterols and peptides	Urethane	-84.99	(93, 95)
		Isothiocyanate	-81.10	
		Dipeptide	-47.36	
		2-Butyloxycarbonyloxy-1(ethanol extract)	-5.583	
		Eicosanoic acid	-3.852	
		Cis-11-eicosenoic acid	-3.00	
*Gynura bicolor*	Sterols	3-Caffeoylquinic acid	-29.07	(123)
		5-O-Caffeoylquinic acid	-27.32	
		3,4-Dicaffeoylquinic acid	-27.17	
		Trans-5-p-coumarylquinic acid	27.11	
*Curcligo latifolia*	Polyphenol Flavonoid Sterol	Phlorizin	-10.9	(104)
		Scandenin	-9.3	
		Mundulone	-9.3	
		Berberine	-8.9	
		Dimethyl caffeic acid	-7.1	
*Melicope latifolia*	Sterols Flavonoids	β-Sitosterol Halfordin Methyl-p-coumarate Protocatechuic acid	Maximum binding energy was of compound 3 (-5.7)	(105)

Among many other medicinal plants, *Terminalia arjuna* is a natural DPP-IV inhibitor with significant cardio-protective effects owing to the presence of some active ingredients, including arjungenin, ellagic acid, and arjunic acid. These active compounds show superior DPP-IV inhibitory activity compared with synthetic inhibitors (64). Nowadays, combinations of ethnomedicine are used, and their efficacy is enhanced by adding bioactivity-determining related ingredients—for example, the efficacy of some of the tea extracts is enhanced by adding bioactive compounds like epigallocatechin-3-O-gallate, kaempferol rutinoside, myricetin-3-0-glucoside, and theogallin. The ethanolic extract of *Eucalyptus citriodora* has active compounds such as rhodomyrtosone B, rhodomyrtosone E, and quercitroside. These compounds have remarkable *in vivo* and *in vitro* effects on managing hyperglycemia. These biochemical compounds enhance insulin functionality in 3T3-L1 cells by improving plasma insulin, glucose tolerance in the high-fat-diet-fed obese rats, and attenuating plasma DPP-IV with a concomitant rise in GLP-1 levels (142). Moreover, a hot water extract from *Heritiera fomes* possesses some bioactive compounds similar to quercetin. These compounds exhibit antidiabetic action, proved using BRIN-BD11 cells and high-fat-fed rats. A significant improvement in glucose tolerance and plasma DPP-IV was observed in rats fed with the abovementioned compounds. It also led to a decrease in intestinal disaccharidase activity while increasing the GI tract motility and transit time (143). In one of the experiments done on the quinine tree (*Rauvolfia caffra sond*), the efficacy of crude extract was found to be exterminated because of the dwindling alliances between alkaloids and saponins. Assessing fractions containing saponins and alkaloids revealed decreased antioxidant and DPP-IV activity. Based on these results, alkaloids and saponins should be kept separate during drug formulation (144).

Using bioactive compounds alone or in combination for their synergistic effects is thoroughly studied for their effectiveness against free radicals. The synergism and antagonism of two potential compounds of the crude extract are well known to be strongly influenced by the structure and spatial conformation of the antioxidants involved. It has been stated recently that several compounds can have synergistic effects in particular ratios, as at a particular concentration one bioactive compound can enhance the bioavailability of the other bioactive compound. With an increase of weaker antioxidants like hydroquinone and resveratrol, synergism decreases. Maximum synergism was observed when rutin hydrate was paired with resveratrol in 3:1 proportion, whereas maximum antagonism was observed when rutin hydrate was paired with hydroquinone in 1:3 proportion (145, 146). Moreover, studies have demonstrated that the efficacy of different combinations is also influenced by molar ratio, and the externalization of synergy strongly depends on the inflow of free radicals (147). In one of the studies, the combination of *Terminalia arjuna* and *Commiphora mukul* exhibited synergism and augmented antioxidant activity as measured by the normalization of superoxide radicals and nitric oxide levels (148).

Moreover, several polyherbal formulations like BGR-34 (blood glucose regulator) were marketed by Aimil Pharmaceuticals, which was approved by the AYUSH ministry, Government of India. It was named after 34 different active phytoconstituents formulated with a consortium of six different plant extracts, including the stem extract of *Berberis aristata*, *Pterocarpus marsupium*, and *Tinospora cardiofolia*, the leaf extract of *Gymnema sylvestre*, and the seed extract of *Trigonella foenum graecum.* It has exhibited DPP-IV action along with its cardioprotective effects, reduced glycated Hb level, enhanced antioxidant action, and regulated glucose homeostasis (124, 149, 150). Similarly, Insulin Management Expert-9, which was developed by the Central Council of Ayurvedic Sciences (CCRAS), is a consortium of five different plant extracts, including the seed extract of *Mangifera indica* and *Syzigium cumini*, the fruit extract of *Momordica charantia*, the leaf extract of *Gymnae sylvestre*, and the exudates of *Asphaltum punjabinum.* Its effects led to the regeneration of pancreatic β-cells, stimulation of insulin production, decrease in insulin resistance, delayed insulin resistance, delayed intestinal absorption, and reduced sugar cravings (124, 149, 150).

**Figure 6:**
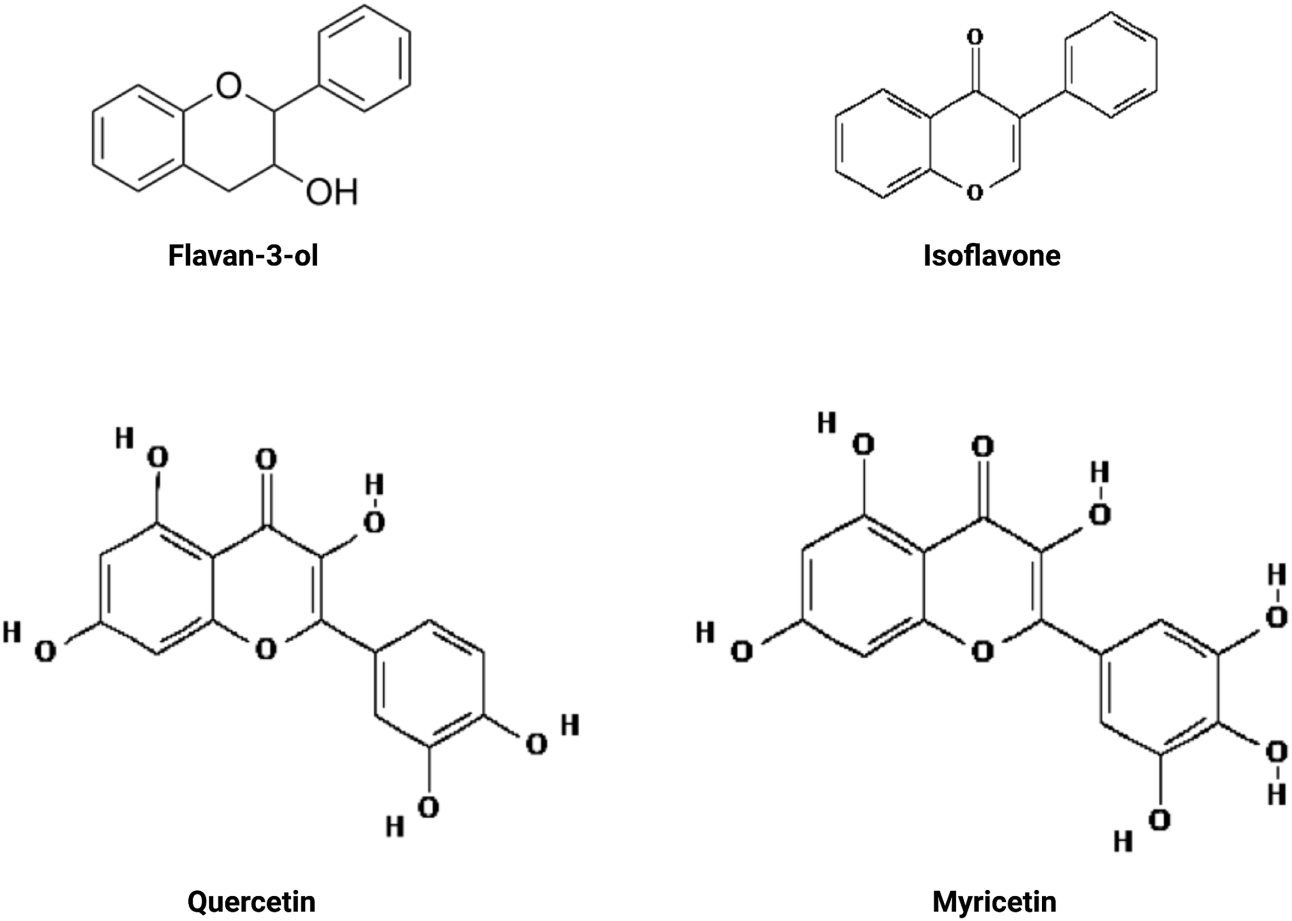
Flavonoids exhibiting an association with a reduced risk of type 2 diabetes mellitus in clinical studies.

Flavonoids (**Figure 6**) are the most abundant plant polyphenolics that are consumed on a regular basis by humans, which can modulate DPP-4 activity, thus exerting their anti-diabetic effects (115). Whether key flavonoid subclasses (anthocyanins, flavones, flavonols, flavanones, and flavan-3-ols) in the diet are connected with the incidence of T2DM has been the subject of a number of research. Several randomized clinical trials (RCTs) have demonstrated the importance of flavonoids in T2DM, especially in long-term trials, but short-term therapies had no discernible impact on blood glucose levels or insulin resistance. An 8-week RCT by Hall et al. (2006) (151) and a 6-month RCT using soy protein and isoflavones did not find a beneficial effect on the plasma concentrations of insulin and glucose in postmenopausal women (152). In another study, green tea extracts containing 456 mg of catechins (a type of flavonoid) daily did not have noticeable effects on blood glucose levels or insulin resistance in an RCT lasting for 2 months (153). However, the long-term randomized, double-blind, placebo-controlled clinical investigation indicated the benefit of green tea extract on patients with T2DM and lipid abnormalities. For the duration of the 16-week research, the therapeutic group took 500 mg of green tea extract three times daily, whereas the control group received cellulose at the same dose and frequency. An increase in GLP-1, likely by inhibiting DPP-4, and a decrease in triglycerides and insulin resistance were noticed in the therapeutic group who received green tea extract (154). Additionally, Curtis et al. (2012) reported that postmenopausal women with T2DM who took flavan-3-ols and isoflavones for a year exhibited significant reductions in estimated peripheral insulin resistance and persistent improvements in lipid profile and insulin sensitivity (155). Similarly, a meta-analysis of 24 RCTs by Shrime et al. (2011) concluded that consuming flavonoid-rich cocoa significantly improved insulin resistance (156), and a meta-analysis of the effects of cocoa, chocolate, and flavan-3-ols from 42 RCTs was summarized by Hooper et al. (2012), who noticed significant reductions in insulin resistance and fasting serum insulin after cocoa or chocolate interventions (157). Notably, flavan-3-ols are among the most abundant bioactive components and account for 82.5% of the total flavonoid consumption among adults in the United States (158).

While the consumption of anthocyanin-rich foods, notably blueberries and apples/pears, was related to a decreased risk of T2DM in US men and women, no significant relationships were reported between total flavonoid intake and other flavonoid subclasses and T2DM risk (159). Dietary intakes of the flavonoids quercetin (found in fruits and vegetables) and myricetin (found in tea, berries, fruits, vegetables, and medicinal herbs) were found to be marginally associated with a reduced risk of T2DM in a study involving 10,054 Finnish men and women (160). Because of the contradictory results, well-designed, long-term studies are required to determine whether flavonoids offer protection against T2DM.

## Discussion

Since antiquity, natural product leads have been enriched with bioactive compounds encompassing a huge arena compared with small synthetic molecules (161). Myriads of bioactive compounds have made an incredible contribution to pharmacotherapy. They are far superior to structural leads based on diversified chemical properties. The presence of structural complexity and scaffold diversity is the most distinguishing and prominent feature which has characterized natural drugs to be better and more advantageous than synthetic drugs (162, 163). The existence of higher molecular mass, a larger number of sp3 carbon atoms, more oxygen atoms, and lesser nitrogen and halogen atoms serve to be hydrogen donors and acceptors with greater molecular rigidity over synthetic compounds. Earlier synthetic drugs were preferred over herbal ones because of some shortcomings. To isolate and identify bioactive compounds from plant origin is a challenging task as plants can have a varied chemical composition influenced by various parameters like environmental conditions and choice of extraction methods (164) which can create obstructions during isolation. Henceforth, new advanced technologies have been introduced to reduce the risk of degradation of plant extracts. Microwave, ultrasonic-assisted, and accelerated solvent extraction have outcompeted and flourished over conventional techniques. Currently, the application of ionic liquids, also known as designer solvents, have been used for MAE as well as UAE, have been strongly preferred over other classical solvents for their novel properties, and have replaced several volatile organic compounds in solvent extraction (165). Recent findings have also suggested that adapting the supercritical fluid extraction methodology for extracting various natural products results in better yield (164, 166). As a deduction, herbal leads are perpetuated with a humongous repository of compounds to delineate various pharmacognostic products. With evolving times, people are getting more inclined towards adopting a healthy lifestyle and are required to get nutritionally enriched products with more antioxidants and bioactive compounds; sometimes, these are lost while undergoing processing. Hence, improvement of physicochemical properties is needed along with retention of phenolic compounds by maintaining all the organoleptic characteristics in the final product. Therefore, many emerging treatments are needed to preserve the characteristics of the food product, and by following the block freeze crystallization (BFC) technique, the entire solution is subjected to a freezing process, which is further thawed by microwave heating with separation and extraction of cryoconcentrated solution by centrifugation and vacuum from the frozen matrix. Maintaining concentration is the major trump card of this technique. An advancement of adding a filter to centrifugal BFC is to increase solute efficiency and solute yield. While undergoing experiments with green tea extract, the number of phenolics and antioxidant concentrations was increased by following the BFC technique (167, 168). Moreover, it is well established that obtaining a powder form of pomegranate fruit by performing BFC and spray drying resulted in greater retention of antioxidants as well as significant increases in physical parameters such as solubility index, bulk density, and hygroscopicity (169). The previous studies conducted on fruits of *Citrus sinensis* demonstrated that freeze drying technique expressed a higher content of phenol and also antioxidants. Flavonoids (hesperidin) were found to be decreased during heat drying (170). Due to the heat-sensitive nature of phenol compounds and the activation of polyphenol oxidase and peroxidase during the thermal drying process, phenolic compounds are destroyed. In contrast, polyphenol oxidase enzyme activity was reduced in freeze drying, which was conducted at a lower temperature. So, instead of using a heat drying process, freeze drying is the best alternative for preserving and quality extraction of bioactive compounds (170). A study on the preservation of antioxidant capacity and health-promoting components in frozen baby mustard also found that blanching before freezing diminishes antioxidant capacity levels as well as the contents of high-content glucosinolates and ascorbic acid (171).

With the recent progression in advanced drug discovery, several *in silico* approaches have been re-strategized for testing and synthesizing new phytochemicals as anti-diabetic compounds (172). Moreover, in terms of pharmacological and pharmacokinetic synergism, polyherbal formulation offers a better therapeutic effect compared with a single multi-component drug; hence, this strategy produced maximum inhibition with minimal adverse effects (124). Although phytochemicals have gained much interest as therapeutic negotiators, a gap exists between the *in vitro* examination and *in vivo* outcomes, eventually impeding clinical performance. To compensate for its efficacy in this regard, the application of nanotechnology has proven to be the best approach by turning all the odds associated with their pharmacokinetic profile. In accordance with the same from recent findings, nanocarrier-assembled nanoparticles fabricated with bioactive compounds act as hypoglycemia agents, which, in turn, strongly influence the effectiveness of the anti-diabetic agents to improve drug penetration within the GI tract against the specific target, perpetuating the hypoglycemic effect with minimal side effects (173). In line with the discussion above about DPP-IV inhibition by the methanolic and aqueous extract of *U. lobata*, it was understood that methanolic extract expressed better *in vitro* results than aqueous extract. However, on the contrary, aqueous extract expressed better *in vivo* results, but not methanolic extract because of lack of solubility and bioavailability within the GI tract, and to improvise the efficacy, the methanolic extract could be fabricated with the nanoformulations for enhancing the bioavailability within the GI tract.

In summary, the DPP-IV inhibitory properties of phytochemicals have sparked an interest in their potential role in glycemia management. Results from *in vitro* and animal research highlight the potential of these natural inhibitors as plant-based constituents to complement pharmacotherapy in the regulation of blood glucose levels; however, human studies on these compounds are limited. The next stage in researching plant-derived DPP-IV inhibitors is to gather crucial data on the bioaccessibility and bioavailability of these bioactive compounds as well as clinical proof of their usefulness in humans and probable **Table 4** interactions with presently prescribed medicines. DPP-IV inhibitors derived from plants may be developed as dietary supplements to minimize the risk of developing hyperglycemia or they may be used with anti-diabetic medications currently approved for glycemic management in T2DM; however, additional research is required to determine the strategies that will result in economically producible levels of these inhibitors.

**Table 4:** Different marketed polyherbal drugs with their composition and mechanism of action as dipeptidyl peptidase-IV inhibitors.

Plant	Failed antidiabetic action of plant extracts	Nanophytomedicine: mode of action	Reference
*Leonotis leonurus* (,ajor phytoconstituent: marrubin)	*In vitro* experiments were performed on Chang liver cells and INS-1 cells exposed to hyperglycemic conditions. The extract was able to induce insulin secretion and upregulate the GLUT-2 gene The extract could not portray its effectiveness in the gastrointestinal tract because of its big size and poor solubility and bioavailability	Leaf extracts fabricated with homogenized nanolipid carriers demonstrated its efficacy on INS-1 pancreatic cells and changed the liver cells exposed to hyperglycemic conditions by improving the size of the formulation to increase the solubility and bioavailability	(131)
*Ficus religiosa*	Insulin-sensitizing action and hypoglycemic action were reduced	Solid lipid nanoparticles fabricated with extract induced hypoglycemic effect insulin secretion	(174)
*Momordica Charantia* (major phytoconstituent: alpha eleostearic acid)	The bitter gourd oil enriched with conjugated linolenic acid is known to increase the antioxidant activity of enzymes within *in vivo s*ystems Deprivation of bioavailability in the gastrointestinal tract was a constraint which inhibited its efficacy against reactive oxygen species	Since bioavailability was a constraint, hence, to upskill extract formulation, henceforth oil-based nanoemulsions were formulated with low dosage, maximum bioavailability, and efficacy against reactive oxygen species	(135)
*Argyeria nervosa*	The aqueous leaf extract has proven to be a potent antidiabetic formulation for the presence of polyphenols The extracts expressed limited efficacy against the target enzymes because of poor solubility	With an intention to obtain maximum antidiabetic efficacy and effectiveness against free radicals, functional groups of different phytochemicals were coupled to a silver nanoparticle surface in order to reduce the surface area and to obtain maximum antidiabetic activity	(175)
*Eysenhardtia polystachya*	The methanolic and aqueous extract prepared is enriched with an abundance of flavonoids to combat diabetic complications The plant extract showed its limited efficacy because of poor bioavailability within the gastrointestinal tract	With a rationale to improve and showcase antidiabetic effects, extracts are fabricated with nanoparticles to ameliorate insulin resistance and hyperglycemia in INS-1 cells and zebra fish, respectively, by increasing the bioavailability with minimal dosage	(130)
*Marsilea quadrifolia*	The plant extract was found to be enriched with phenolics and flavonoids which could impart its antidiabetic property The extract with poor bioavailability impeded glucose availability to 3T3L adipose cells	Flavonoids and polyphenols of the extract attached to the surface of AuNPs improved the bioavailability and induced transmitted GLUT -4 vesicle to the cell membrane and its uptake in adipocytes Functional groups of different phytochemicals coupled to gold nanoparticles resulted to the formation of biogenic gold nanoparticles, which further reduced the surface area by improving the cellular viability and glucose availability in 3T3 adipocytes	(176)
Oat derived peptides	Peptides is potent enough to inhibit DPP-IV inhibitory activity, but these peptides are degraded by gastrointestinal tract hydrolysis	Nanocrystallization of solid nanoparticles was undertaken in order to protect the oat-based bioactive peptides in a simulated gastric fluid environment with a view to inhibit dipeptidyl peptidase-IV	(129)

## Concluding remarks

At the molecular level, DPP-IV inhibitors have proved their functional role by holding an important position in drug discovery by hampering the deterioration of incretin hormones and further preserving their endogenous and blood glucose levels. Additionally, it also aids in the regeneration of pancreatic β-cell mass and enhances skeletal muscle cell proliferation. Nowadays, pharmacognosy has flourished enough to design herbal drugs far more reliable and superior to synthetic drugs with the least adverse effects. This review article aims to shed some light on the potency of different isolated bioactive compounds, polyherbal formulations, nanophytomedicines, and their functional role in efficacious treatment for T2DM. With anticipation of promoting pharmacognosy and phytochemistry to young researchers, this review is entailed with all the information which will be a steppingstone to designing and developing an herbal drug against the target enzyme to maintain endogenous incretin levels.

## Author contributions

Original concept, writing the original draft, editing, and methodology: SC. Writing the original draft, editing, and methodology: SM. Writing the original draft, editing, and methodology: SV. Writing the original draft, editing, methodology, and proofreading: DS. Writing the original draft, methodology, and proofreading: PM. Writing the original draft, editing, methodology, and proofreading: BP. All authors contributed to the article and approved the submitted version.

## Funding

Schemes [number ECR/2016/001984 by SERB, DST, Government of India, and 1188/ST, Bhubaneswar, dated 01.03.17, ST-(Bio)-02/2017, DST, Government of Odisha, India] to BP are acknowledged.

## Acknowledgments

The support from Iowa State University, President Science College, and St. Xavier’s College, Gujurat, India, and from Odisha University of Agriculture and Technology, respectively, are highly acknowledged.

## Conflict of interest

The authors declare that the research was conducted in the absence of any commercial or financial relationships that could be construed as a potential conflict of interest.

## Publisher’s note

All claims expressed in this article are solely those of the authors and do not necessarily represent those of their affiliated organizations, or those of the publisher, the editors and the reviewers. Any product that may be evaluated in this article, or claim that may be made by its manufacturer, is not guaranteed or endorsed by the publisher.
